# Barriers and facilitators to implementing addiction medicine fellowships: a qualitative study with fellows, medical students, residents and preceptors

**DOI:** 10.1186/s13722-017-0086-9

**Published:** 2017-09-20

**Authors:** J. Klimas, W. Small, K. Ahamad, W. Cullen, A. Mead, L. Rieb, E. Wood, R. McNeil

**Affiliations:** 1 Department of Medicine, B.C. Centre on Substance Use, St. Paul’s Hospital, University of British Columbia, 608-1081 Burrard Street, Vancouver, BC V6Z 1Y6 Canada; 2 Department of Family Practice, University of British Columbia, 1081 Burrard St., Vancouver, BC V6Z 1Y6 Canada; 3 Department of Family and Community Medicine, St. Paul’s Hospital, 1081 Burrard St., Vancouver, BC V6Z 1Y6 Canada; 4 School of Medicine, Coombe Healthcare Centre, University College Dublin, Dolphins Barn, Dublin 8, Ireland; 5 Faculty of Health Sciences, Simon Fraser University, Blusson Hall, 8888 University Drive, Burnaby, BC V5A 1S6 Canada

**Keywords:** Addiction, Substance-related disorders, Medical education, Qualitative research

## Abstract

**Background:**

Although progress in science has driven advances in addiction medicine, this subject has not been adequately taught to medical trainees and physicians. As a result, there has been poor integration of evidence-based practices in addiction medicine into physician training which has impeded addiction treatment and care. Recently, a number of training initiatives have emerged internationally, including the addiction medicine fellowships in Vancouver, Canada. This study was undertaken to examine barriers and facilitators of implementing addiction medicine fellowships.

**Methods:**

We interviewed trainees and faculty from clinical and research training programmes in addiction medicine at St Paul’s Hospital in Vancouver, Canada (N = 26) about barriers and facilitators to implementation of physician training in addiction medicine. We included medical students, residents, fellows and supervising physicians from a variety of specialities. We analysed interview transcripts thematically by using NVivo software.

**Results:**

We identified six domains relating to training implementation: (1) organisational, (2) structural, (3) teacher, (4) learner, (5) patient and (6) community related variables either hindered or fostered addiction medicine education, depending on context. Human resources, variety of rotations, peer support and mentoring fostered implementation of addiction training. Money, time and space limitations hindered implementation. Participant accounts underscored how faculty and staff facilitated the implementation of both the clinical and the research training.

**Conclusions:**

Implementation of addiction medicine fellowships appears feasible, although a number of barriers exist. Research into factors within the local/practice environment that shape delivery of education to ensure consistent and quality education scale-up is a priority.

## Background

Around the globe, harms stemming from substance use represent a significant social, health, and economic burden [1]. The associated mortality and morbidity stemming from substance use (e.g., HIV, hepatitis C) place considerable demands on healthcare systems [2, 3] and represent an urgent public health priority. Advances in addiction science have helped to identify effective treatments for substance use disorders (e.g. opioid agonist therapies, contingency management) [4, 5]. These treatments are often delivered in general medical settings and are associated with significant improvements in health and social outcomes of people with substance use disorders (SUD) [6, 7], including physical and mental health functioning [8].

The important role of physicians in the management of SUD is well documented [9, 10]. Specifically, evidence-based therapeutic interventions delivered by trained physicians, including pharmacological and psychosocial interventions, can increase motivation for and enrolment in specialised treatment programmes [11]. For example, people receiving opioid agonist treatment in primary care are twice as likely to stay in treatment compared with those who attend a specialist site [12]. However, the impact of physicians in SUD-related care is often diminished due to the widespread underutilisation of evidence-based treatments for SUDs [13].

Adequate diagnosis and treatment of SUDs by physicians often does not occur due to a lack of knowledge and accredited training in addiction medicine [14, 15]. Historically, undergraduate medical education and postgraduate clinical training programs have not invested in the implementation of addiction medicine training for health care providers, and, when they have, it has mostly been for psychiatrists trained in small programmes [13, 16]. As a result, many physicians feel unprepared to treat people with SUDs, most of whom receive care from non-medical professionals without formal substance-related training [13, 17]. Recently, a number of diverse initiatives to address this shortcoming have emerged internationally. For instance, the Addiction Medicine Foundation (AMF) has established fellowships in addiction medicine and accredited 27 of these programmes (63 total slots annually) to date, including four programmes (16 slots) in Canada [18]. This limited number of training opportunities falls far short of the demand for specialised addiction treatment services due to the high number of people with SUDs who need such treatment [1]. Countries like Australia or Netherlands have developed substantial training programmes and Masters in Addiction Medicine, respectively [19]. Other governments (e.g., Norway) have recognised the increasing interest in addiction medicine among doctors and created addiction medicine diplomas or specialties [19, 20]. Focusing on the new generation of doctors, the UK’s project on ‘Substance Use in the Undergraduate Medical Education’ improved the addiction medicine knowledge of medical students [21], while the importance of addiction medicine training for clinicians has also been recently highlighted in Ireland [22]. Unfortunately, although these programmes teach addiction medicine to physicians, their content and intensity varies significantly from country to country.

To overcome the deficits in training locally, two fellowship training programmes have been established in Vancouver, Canada: (1) the interdisciplinary St. Paul’s Hospital Goldcorp Addiction Medicine Fellowship, and (2) the Canadian Addiction Medicine Research Fellowship [23]. Of note, Vancouver has Canada’s largest drug scene, which has been a significant driver of local HIV and hepatitis C epidemics [24]. As a result, this has led to an environment in which drug policies and programmes have been launched as pragmatic responses to the local drug use epidemic (requiring comprehensive responses) and their successful evaluation has led some to be adopted or pursued elsewhere [25]. The two fellowships are examples of such pragmatic responses.

First, within this environment operates the St. Paul’s Hospital Goldcorp Addiction Medicine Fellowship that provides 12 months of funded training to 12 trainees from Psychiatry, Internal Medicine, Family Medicine, Social Work and Nursing. The physician component is accredited by the AMF and includes specialty training in in-patient and outpatient addiction management, as well as concurrent disorders [26]. There are nine core mandatory blocks of four weeks’ duration each, and three elective blocks. The core blocks are: (1) the St. Paul’s Hospital Addiction Medicine Consultation Service; (2) inpatient and outpatient chemical dependency detox; (3) outpatient chemical dependency; (4) women’s recovery; (5) pain management; (6) management of concurrent disorders; (7) inner city youth mental health programme; (8) longitudinal outpatient continuity of care experience, and (9) research. Fellows’ salary is funded through a private donation and the B.C. Ministry of Health. For further description of how the programme is delivered, please refer to previous publication [27].

Second, a new research fellowship for addiction specialists was launched in 2014. The Canada Addiction Medicine Research Fellowship trains physicians to develop the skills required for a career as clinician-scientists in substance use research. This training occurs through: (1) immersion in SUDs research training programme (i.e., British Columbia Centre on Substance use and B.C. node of the Canadian Research Initiative in Substance Misuse); (2) training in diverse research methodologies (e.g., cohort studies, qualitative studies) through didactic lectures, workshops, and monthly journal clubs; (3) mentorship in the development of manuscripts for submission to peer reviewed journals using data from two prospective cohorts of people who use drugs [28–30]. Each year, four part-time, one-year fellowships of $50,000 CDN each are available thanks to funding from the National Institute of Drug Abuse. The content and delivery methods of the fellowship have been described elsewhere [31].

Finally, the Addiction Medicine Consult Team (AMCT) at St. Paul’s Hospital supports the fellowship programmes and is a distinct clinical service consisting [26]. AMCT provides inpatient Addiction Medicine consultations to general inpatient and psychiatry wards in the hospital. Patients come often from the Downtown Eastside area of Vancouver, BC, where AMCT’s colleagues from the B.C. Centre on Substance Use conduct longitudinal cohort studies of people who inject drugs or who live with HIV/AIDS. The overlap between research and clinical care informs research agendas and fosters the uptake of novel research findings in practice [26, 32]. In sum, the integration of both research and clinical training in addiction medicine at the under- and post-graduate level, which has been developed within a single academic centre, is unique and has not been described previously. We sought to develop a more complete description of the implementation process to aid educators and administrators in the development of similar programmes elsewhere [33].

We, therefore, conducted a qualitative evaluation of this rare combination of clinical plus research training courses, focusing on barriers and facilitators of implementing physician training in addiction medicine.

## Methods

We conducted qualitative interviews to explore implementation of the St. Paul’s Hospital Goldcorp Addiction Medicine Fellowship and the Canada Addiction Medicine Research Fellowship, as well as barriers and facilitators to the implementation of these fellowship programmes. We selected the qualitative design specifically because of its capacity to elucidate participants’ experiencing during the implementation of these fellowship programmes and thus deepen understandings of contextual influences on their uptake [34, 35].

We sought to recruit individuals who: had competed a clinical fellowship, research fellowship, or enhanced skills training; were staff of the AMCT; and, had completed a 1-month research rotation with the training programme as part of their undergraduate medical training or residency. We also sought to recruit (4) teaching faculty for the fellowship (including nurse, social worker and fellowship director). We sent an email to all potential participants explaining the study and inviting them to participate. Two email reminders followed if they did not respond between March and July 2015. We based our interview guide on a scoping literature review about addiction medicine education and a qualitative study on a similar topic that piloted the questions [36, 37]. The first author conducted and audio-recorded the interviews in the hospital, or in a location convenient for participants; external staff transcribed the recordings. All participants were informed of the study purposes, voluntary and confidential participation, before they signed informed consents.

Data were imported into NVivo (version 10), a qualitative data analysis software programme, to facilitate coding. We analysed the data according to Braun and Clarke’s five-step process, including: (1) data preparation, transcription and familiarization; (2) generation of initial codes; (3) theme assessment; (4) theme review; and, (5) theme finalization [38, 39]. Furthermore, our analysis was informed by Damschroder et al.’s [40] Consolidated Framework for Advancing Implementation Science Research (CFIR). This meta-framework attempts to unify all published implementation theories based on the robustness of the evidence behind them. As such, its generic nature allows studying underlying concepts to overcome artificial barriers and to transcend beyond the limitations of individual “labels”. The framework has five major domains: intervention characteristics, outer setting, inner setting, characteristics of the individuals involved, and the process of implementation [40]. The first author analysed the data, and two team members reviewed data and provided feedback on the analysis and themes.

## Results

### Participant demographics

In total, 26 learners from the 2013–15 training cohorts (84% of 31 potential participants) participated in this study, including 14 women and 12 men. All participants were involved in the fellowship programmes as learners (n = 23) or staff (n = 3). Participants included: (a) clinical fellows (*n* = 8); (b) research fellows (*n* = 4); (c) enhanced skills learners (*n* = 2); (d) students and residents who had completed a 1-month rotation and prepared a case report or other publication (*n* = 11); and, (e) staff of the AMCT and teaching faculty for the fellowship (including nurse, social worker and fellowship ex-director; *n* = 4).

**Fig. 1:**
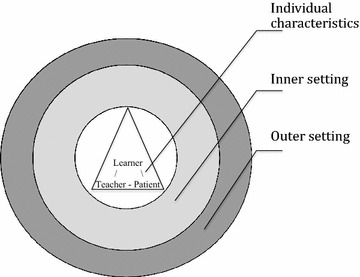
Framework for implementation of addiction medicine education. *Note* At the heart of the training implementation was the learner-mentor-patient triad set in the organisational and structural context. We operationalized the outer setting as structural, community and organisational concerns, the inner setting as learner concerns, and the individuals involved were teachers and patients [40]. At the individual level, access to the “giant brains” of preceptors fostered learning. At the inner level, it was evident that our learners rose to the challenge of managing their time and balancing competing priorities with their learning. This inner motivation stemmed from personal values and attitudes, which, in turn, were shaped by the community of learning and practice—the final, outer level of implementation

We organised the data in relation to Damschroder et al.’s consolidated framework into six major types of barriers and facilitators of the implementation: (1) structural, (2) organisational, (3) mentor, (4) learner, (5) patient and (6) community concerns. As shown in Fig. 1, at the heart of the training implementation was the learner-mentor-patient triad set in the organisational and structural context. We operationalized the outer setting as structural, community and organisational concerns, the inner setting as learner concerns, and the individuals involved were teachers and patients.

## Structural concerns

### Funding for the training helps “get rid of the fire” but not completely

Although funding for the fellowship programmes was welcomed, it was perceived as a partial solution in efforts to address the underlying conditions affecting people with SUDs. For example, SUDs were characterised by one of the participating physicians as “the smoke from a fire, and the fire is burning really strongly right now, and the fellowship is a way to train fire people, although you need more than just a fire person to put out a fire. [Participant #24]” She further emphasized that the training is an important aspect of solving SUDs. However, as she explained, it is not the ultimate answer:

“It’s [fellowship] just going to make a dent in getting rid of that fire [SUD], and it’s an important aspect of it, and it’s great that people are getting opportunities to grow and change and focus on this and learn about all the different nuances of addiction medicine etc., but it’s not [the answer].” [Participant #24, clinical fellow]

Most of the patients treated by study participants in the St Paul’s Hospital were extremely marginalised people with multiple chronic diseases, were despised by the mainstream society and engaged in shunned income-generation activities that included scavenging and stealing. While quality health care provided by qualified professionals can improve health of people with SUDs, it cannot in and of itself fully address the underlying issues of poverty, displacement, colonisation, homelessness, and unemployment.

Faculty and administrative staff perceived the funded fellowship programmes (full or partial) favourably because it secured protected time to build the educational infrastructure of the Addiction Medicine Fellowship (e.g., clinical sites for rotations, didactic sessions and materials). From the learners’ perspective, the funding allowed them to engage in learning activities and limit clinical duties:

“It was an opportunity where you could be funded part-time to step away, a little bit away, from clinical responsibilities.” [Participant #1, research fellow]

The funding also accelerated the fellowship establishment by providing financial stability and allowing the accreditation of the fellowship, giving fellows the opportunity to apply for the license from the AFM, and thus supporting the growth of the SUD specialist workforce. For example:

“Then, funding came in the summer of 2012 which really again boosted us a lot cause we knew it could be a reality, and then we applied for [accreditation].” [Participant #11, faculty]

### Implementation of knowledge and practice environment and patient population

The learners recognised that the fellowship “really was geared to teaching the science behind addiction.” However, the ‘knowledge’ learned through the fellowship was not always perceived as transferable to daily practice because of the nature of practice environment and patient population. Therefore, it was necessary to adapt practices to the specifics of the environment and population, as well as broader social-structural determinants of health (e.g., insurance, employment). Some participants saw potential financial constraints as a barrier to treatment provision, especially among low-income populations. As one participant explained:

“I had difficulty because I knew that none of the patients that I would end working with would be able to even afford [these specific medications].” [Participant #10, clinical fellow]

The preceptors applied best-practice guidelines in their decisions intuitively without talking to learners about the evidence, or specific trials, explicitly. The following quote illustrates barriers encountered by the participants when implementing new knowledge and the iterative process of seeking new evidence and applying it in practice:

“I don’t [think] it’s always verbalized that we’re choosing this medication because this is the evidence-based medication, it’s just kind of get [it] done and then you sort of have to figure out later whether that was the most correct decision…” [Participant #16, resident]

She continued to describe financial and social barriers to implementing the learning on evidence-based medicine in disadvantaged populations:

“There are limitations, we always say there’s no typical patient, especially on the [hospital] addiction service, because there are so many limitations around finances, around social issues that influence people’s […] results, treatment and you can’t always do what might be the best possible thing, because it’s not safe in that situation, or it’s not feasible…” [Participant #16, resident]

She further describes how the patient in question experienced multiple methadone and antiretroviral treatment interruptions and re-initiations due to drug use and social instability. The participant described that the most evidence-based approach in this situation would be to start the patient on an opioid agonist and an antiretroviral treatment, and to keep her on them “forever,” but felt that it might not be “doable” or given the underlying social-structural inequities.

## Organisational concerns

### Organisational and staffing support as the ‘backbone’ of implementation

Participant accounts underscored how faculty and staff facilitated the implementation of both the clinical and the research training. They included not only mentors and administrators, but also attending physicians, statisticians, senior researchers and other centre staff. Senior researchers met with the learners to formulate their research questions, draft analysis plans and refine the manuscripts. Centre staff helped with other tasks, such as, admission, clinical rotations or organisation of meetings. Statisticians analysed the data for the learners’ manuscripts. As one participant spoke about his relationships with the clinical team:

“I’ve actually established nice long-standing relationships with almost everyone who I worked with on the [hospital] addiction service which is fantastic.” [Participant #3, resident]

Participants from both streams—clinical and research—emphasized the utility of the overlap between faculties of both streams that ensured continuity of their learning process. Some learners did the clinical fellowship and then the research fellowship and were then in the programme for two years, maximizing opportunities for learning.

### First-year hurdles—infrastructure and resources

Time constraints and limited availability of research or clinical space were the main barriers in the organisational domain. The learners pointed to the newness of the fellowship that was lacking infrastructure in some rotations (e.g., financial, technical and bureaucratic infrastructure). One or two rotation clinics did not have a learning space with a desk for participants. This prevented people from performing tasks learned in their clinical training:

“[The clinical rotation] was quite disorganised and they didn’t really have much of a teaching infrastructure developed when I went through, so there was a lot of independent work at that rotation. It was ok but there’s areas of improvement for that rotation, for sure.” [Participant #26, clinical fellow]

## Mentorship concerns

### Mentors’ responses

There was considerable overlap between mentors for the clinical rotations, research projects and fellowships that fostered development of working relationships between faculty and learners. It allowed participants to continue their professional growth and move between different educational programmes. Some learners suggested that mentors needed to supervise their work more closely, especially for research projects. Therefore, the main issues within this domain were interpersonal. If mentors met with the learners regularly, learners were able to track their progress better:

“I think if there’s set blocks maybe even just once a month where you have like a half an hour sit-down with the mentors, which should be mandatory, where you can go over the month, the progress, the struggles, what works, what didn’t work - I think that would be helpful.” [Participant #26, clinical fellow]

### Educators looked up to as ‘role models’

Teaching made clinician teachers “better doctors” and their characteristics were paramount in clinical learning through role modelling: “I’m a better doctor because I’m a teacher at the fellowship [Participant #12, faculty].” If the teacher was from the same medical discipline, learners perceived it as being especially helpful. Furthermore, non-physician mentors sometimes induced stress in learners by requesting too many updates. Learners felt better understood by physicians because they “went through the medical school” and saw clinical mentors as role models:

“I think also having him [mentor] who’s done internal medicine residency and we had the same training, so from the clinical aspect, I looked up to him.” [Participant #10, clinical fellow]

## Learner concerns

### Tough balance

Learners’ concerns included barriers and facilitators of programme implementation from the perspective of trainees. The lack of previous background in research among clinicians was perceived as a barrier to training in addiction medicine research. At times, learners coming from more clinical backgrounds felt frustrated, isolated, and anxious about the future, especially in cases where their previous research training was limited. By extension, physicians on clinical rotations struggled with the prevailing stigma associated with drug use. Although they recognised that their peers did not generally see medically managing SUDs as a “super popular thing to do,” they thought that training SUD specialists, and creating jobs for them in health care, could help establish addiction medicine as a respected specialty and counter existing stigma.

For most learners, training in addiction medicine and research was something performed in addition to their already busy schedules, which included seeing patients and running clinics. Providers with high clinical workloads struggled in the clinical and research training activities and some clinical rotations were busier than others. The tension between training and competing priorities is well illustrated in this participant’s quote:

“The one thing that I’m struggling a little bit with is that I’m busier this year than I was last year, and the project to me is a bit bigger as well, so this time, I feel like I’m the one slowing the project down cause I’m not always able to get back to the researchers.” [Participant #8, student]

### Learners prioritise writing papers over “twiddling their thumbs”

Demanding workloads put an increased strain on the participants. However, learners sought to take steps to manage their time effectively and efficiently, such as rotating their tasks or finding some extra time in their schedules. As one learner explained:

“I think always trying to have a challenge on the side so that’s why I was so happy to engage in so many different research projects that year because if there was a couple of hours of down time, I made sure that I had something that I could be doing [Writing papers] yeah exactly, or editing, or whatever as opposed to just sort of sitting here twiddling my thumbs or going for coffee.” [Participant #10, clinical fellow]

Other facilitators of clinical-research training were mainly related to the personal characteristics of learners, such as previous background and training in research and motivation to learn from the experience. Those who were capable of self-directed learning benefitted from the training the most because of the experiential nature of learning. For example:

I feel like I’m able to provide better care, and talk to patients, and educate them around their disease, and I’m more comfortable teaching, once I’ve personally had a bit of experience in it. […] the more cases I see, and the more teaching I do, the more I like it.” [Participant #14, research fellow]

## Patient concerns

### Becoming ‘sensitised’ to learning from patients

Our analysis demonstrated that patients “taught” learners lessons regarding addiction medicine, and thus facilitated learning implementation. Physicians learned that trust in the therapeutic relationship was critical to patient engagement and treatment success. Subsequently, patients’ engagement increased the potential for success of treatment. The physicians became sensitised to learning from patients:

“So, I really learned more and more, just from my participants and the patients that I see.” [Participant #9, nursing fellow]

Having both research and clinical interactions with patients, due to the fluidity between the clinical and science programmes, helped to solidify the new learning:

“It was nice to see that progression where you have an incident and then you can write about it and then let people know that […] It really helped me to appreciate the research.“[Participant #18, student]

However, barriers related mainly to the practice environment and patient population, described above, thwarted this learning. Patients in hospitals had severe SUDs with many concurrent social and mental health problems that rendered them unstable and the complexity of their conditions precipitated numerous challenges related to their care.

### Patients’ struggles

The learners recognised that the patient population in the hospital was more complex than in other settings due to housing issues, mental health comorbidities and polysubstance use disorder that required specialised treatments. The faculty also recognised this dynamic and highlighted the need to de-centralise housing and diversify treatment modalities. Sometimes, the learning was difficult and confrontational, probably varying as a result of setting—inpatient versus outpatient—and help seeking:

“I was verbally assaulted by patients. I had trays hurled at me and I had people who didn’t want to talk about their addictions issues, or receive any sort of care, so that, as the predominant population [in hospital], I found very difficult, whereas an out-patient setting, people are dying to see a doctor for this, and they really wish to get into it, and talk about it, and focus on treatment options.” [Participant #25, clinical fellow]

This experience resonated with the perceived need for outpatient clinical rotation that would give the clinical learners different perspectives. Similarly, the research learners felt their “hands were a bit tied” due to the restrictions integral to the nature of the researcher-participant relationship. Within addiction medicine research, the study restrictions could be difficult to navigate for the clinician-researcher because of other co-morbid diseases and social circumstances that make it hard to just focus on study protocol. As one participant observed during the research interview with a patient:

“I have the best interests of the participant [patient with SUD treated by the service] in mind but within the constraints of a study protocol.” [Participant #16, resident]

## Community concerns

### Gains of the community of practice

The wider context of implementing addiction medicine and best practice was the community of practice [41]. It consisted of colleagues within the healthcare system that were not part of the training, preceptors and staff in the clinical rotations, as well as the prevention and harm reduction organisations not involved in the rotations. This community of practice provided support and mentoring to junior learners, as well as linkages between the senior clinicians and staff. The hospital team was perceived as a group of innovators who sought to provide improved or enhanced care to patients:

“…because I’ve had this contact with them and all so lovely, it’s so easy to have access to these giant brains […] it’s about connection and about creating that web of people that you can use as resources.” [Participant #19, enhanced skills learner]

Although this community was a source of peer support and mentorship, providing many gains for the fellows (e.g., access to experts and expertise or teamwork), being part of it was not without risks.

#### Risks of the community of practice

However, some negative attitudes of this closely woven “web of people” could be detrimental to the growth of an early-career addiction specialist. Some learners were challenged to advocate on behalf of addiction medicine as a discipline because it was seldom considered to be “sexy area of medicine” by colleagues in other disciplines. However, having those conversations forced them to be certain that this was a suitable career path. Other inter-professional challenges within addiction medicine, such as entrenched attitudes and clinical practices, made implementation of new learning difficult:

“I think when people are very set about the way that they should do things. Either because they side with a certain side of the evidence, or if they choose to not follow the evidence, that can make things very difficult because it not only makes the learning difficult, but it also makes discussion and solidification of ideas much more difficult.” [Participant #20, clinical fellow]

## Discussion

Our qualitative analysis of interviews explored how structural, personal and organisational barriers shape the implementation of provider training in addiction medicine. Money, time and space limitations inhibited implementation. Human resources, variety of rotations, peer support and mentoring facilitated training. In summary, our results yield further support for using the Damschroder et al.’s Consolidated Framework for Advancing Implementation Science Research (CFIR) [40] to operationalise and analyse barriers and facilitators of implementing addiction medicine fellowships.

Our participants recalled several formative experiences when their attitude to working with people who have SUDs has been challenged by community members. Although difficult, our findings suggest that having to defend one’s positive regard to working in the SUD field can solidify the resolve of being an SUD specialist [42, 43]. The other CFIR domains of our implementation strategy—intervention characteristics and process of implementation—have been described elsewhere [23, 26].

Several narrative reviews have focused on undergraduate and postgraduate education regarding SUDs [22, 44–46], noting how it is hindered by inflexibility of training programmes and a lack of hands-on training [47–50]. Mentoring in balancing the competing needs of clinical and research careers is inadequate and career guidance is minimal to non-existent [51, 52]. Such an unsupportive training environment can allow physicians to be distracted by other competing interests [49, 53, 54]. Additionally, there seems to be few mechanisms for addiction physicians to pursue formal training in research as clinician-scientists. Programmes, such as the one described in this article, have the potential to overcome these barriers, in addition to integrating addiction medicine into graduate medical education [55]. In particular, the integration should address the two identified major barriers to practicing addiction medicine: (1) insufficient knowledge, training and experience working with patients with SUDs; and, (2) a lack of specialist support [56].

Our results are consistent with previous literature that has endorsed a combined didactic and interactive learning strategy for SUD education [45, 57–59]. Physicians in our study suggested several improvements to the outer level of implementation, especially the structure and organisation of the addiction medicine education. Some suggestions for improvement appeared to reflect the “newness” of the fellowship and that some rotations were having learners present for the first time. This can be overcome by continued funding for the programme and refinement of activities, and subsequent expansion of the SUD-specialist workforce coming out of the fellowship. Indeed, funding current programmes is not enough; new programmes should be established and other comprehensive responses, such as increased profile of SUD and of those who treat it, are needed to meet the needs of people with SUDs. Promoting SUD education among generalist physicians can heighten the chances of screening, early diagnosis and treatment [60]. Although training alone will not solve the SUD problem, it is a *conditio sine qua non* for successful treatment.

There are several limitations to this study. The small sample comprising clinical fellows, residents, students and staff from a single Canadian programme limits potential generalizability. Our participants were not selected randomly, although we invited everybody who was involved in the training and obtained an excellent response rate. We met the threshold of data saturation as recommended for non-probabilistic sample sizes [61]. It is likely that physicians, who seek specialised training, are more likely to have positive attitudes towards, and more clinical experience with, people who have SUD [62]. Nevertheless, the key strength of our study is examination of the unique combination of physician training in addiction medicine and research that provided a rare opportunity to explore the implementation of clinical and academic training in this field. Future studies should truly differentiate the barriers to each type of fellowship program. Though such programs often have common goals, it will be beneficial to more fully understand the challenges experienced by individual programs to further optimize their implementation and impact on learning.

## Conclusion

Training in addiction medicine is feasible and acceptable for healthcare providers. Learners experience the training favourably. Its implementation faces barriers like any other innovation. We must understand the barriers and facilitators specific to these types of programmes if we want to develop stronger local implementation strategies and quality standards. These findings can inspire set up, scale up and standardisation of addiction medicine programmes in other countries.
